# The Emerging Role of Microbial Biofilm in Lyme Neuroborreliosis

**DOI:** 10.3389/fneur.2018.01048

**Published:** 2018-12-03

**Authors:** Enea Gino Di Domenico, Ilaria Cavallo, Valentina Bordignon, Giovanna D'Agosto, Martina Pontone, Elisabetta Trento, Maria Teresa Gallo, Grazia Prignano, Fulvia Pimpinelli, Luigi Toma, Fabrizio Ensoli

**Affiliations:** ^1^Clinical Pathology and Microbiology Unit, San Gallicano Dermatological Institute IRCCS, Rome, Italy; ^2^Department of Research, Advanced Diagnostics, and Technological Innovation, Translational Research Area, Regina Elena National Cancer Institute IRCCS, Rome, Italy

**Keywords:** *Borrelia*, lyme, neuroborreliosis, biofilm, skin, erythema migrans

## Abstract

Lyme borreliosis (LB) is the most common tick-borne disease caused by the spirochete *Borrelia burgdorferi* in North America and *Borrelia afzelii* or *Borrelia garinii* in Europe and Asia, respectively. The infection affects multiple organ systems, including the skin, joints, and the nervous system. Lyme neuroborreliosis (LNB) is the most dangerous manifestation of Lyme disease, occurring in 10–15% of infected individuals. During the course of the infection, bacteria migrate through the host tissues altering the coagulation and fibrinolysis pathways and the immune response, reaching the central nervous system (CNS) within 2 weeks after the bite of an infected tick. The early treatment with oral antimicrobials is effective in the majority of patients with LNB. Nevertheless, persistent forms of LNB are relatively common, despite targeted antibiotic therapy. It has been observed that the antibiotic resistance and the reoccurrence of Lyme disease are associated with biofilm-like aggregates in *B. burgdorferi, B. afzelii*, and *B. garinii*, both *in vitro* and *in vivo*, allowing *Borrelia* spp. to resist to adverse environmental conditions. Indeed, the increased tolerance to antibiotics described in the persisting forms of *Borrelia* spp., is strongly reminiscent of biofilm growing bacteria, suggesting a possible role of biofilm aggregates in the development of the different manifestations of Lyme disease including LNB.

## Introduction

Lyme borreliosis (LB) is the most prevalent vector-borne disease (1) caused by the spirochete *Borrelia burgdorferi*. This Gram-negative bacterium is an obligate pathogen, transmitted to different hosts by ticks in the genus *Ixodes*. LB is frequently reported in North America, Europe, in different parts of Asia, including Mongolia and China as well as in Australia and in Africa (2–4).

LB affects multiple organ systems, including the skin, eyes, joints, muscles, cardiac, and nervous system, presenting, at different stages, with a variety of clinical manifestations (5). Incubation varies from 3 to 32 days, after which a characteristic skin rash, known as erythema migrans, appears in association with fever, headache, malaise, and myalgias (stage 1) (6). After several weeks to months, in 8–15% of patients can be reported the presence of neurologic and cardiac abnormalities (stage 2). Within few weeks, in untreated patients or in case of delayed antibiotic treatment, the infection can disseminate leading to systemic inflammation (7, 8). In the last phase of LB (stage 3), patients may experience chronic monoarticular or oligoarticular arthritis, involving large joints, particularly the knee (9–11).

The most severe manifestations of LB, is Lyme neuroborreliosis (LNB), reported in 10–15% of individuals with localized erythema migrans (12, 13). The activation of the inflammatory response in LNB contributes to the pathogenesis of a broad spectrum of neurologic disorders. Different geographical distribution of *B. burgdorferi* species correlates with specific manifestation of LNB, which is more frequent in Europe than in the North America (6). The most common symptoms of LNB in Europe are painful meningoradiculitis known as Bannwarth syndrome and facial nerve palsy (14). Symptoms involving the central nervous system (CNS) are less common and their exact incidence is not known. *B. burgdorferi* infection of the CNS cause mainly encephalitis, segmental myelitis, cranial neuritis, radiculoneuritis, vasculitis, and intracranial hypertension (13, 15, 16). The clinical manifestation of the LNB may include ataxia, paraparesis, sphincter dysfunction, Parkinson-like symptoms, confusion and cognitive impairment (17, 18). Ischemic stroke is the most frequent cerebrovascular manifestation of LNB presenting in 76% of cases, followed by transient ischemic attack (11%) (19).

LNB is, in many cases, responsive to appropriate antimicrobial therapy and the clinical improvement sustained by the antibiotic treatment provide further evidence for the direct contribution of *B. burgdorferi* in disease pathogenesis. However, the chronic persistence, the frequent reoccurrence of LNB and the ability of *B. burgdorferi* to tolerate multiple cycles of antibiotic treatment is strongly suggestive for the formation of biofilm or biofilm-like protective structure (20–23). Indeed, different studies have shown that *B. burgdorferi* can switch from a motile to a stationary status, in which the cells are embedded within a biofilm matrix (22). *B. burgdorferi* biofilms have been observed both *in vitro* and in human infected skin tissues (22, 23). These structures express different mucopolysaccharides, particularly alginate, extracellular DNA and calcium, which are all typical markers of biofilm (22). The presence of biofilm may explain the low rate of *Borrelia* detection in the blood of infected patients as well as the ability of the spirochetes to evade the host immune system and resist the antibiotic therapy (21, 24–27).

This review investigates the differences in the epidemiology and clinical manifestations of LNB with particular emphasis on the pathogenetic role of *B. burgdorferi* biofilm in tissue adhesion, colonization and survival.

## Materials and methods

The present review focuses on a systematic review of the literature to identify all published articles of LNB using online databases (PubMed, Web of Science, and Google Scholar). The reference list was updated in September 2018. There were no language restrictions; The search terms were “*Borrelia*,” “*B. burgdorferi*,” “*Borrelia* biofilm,” “Lyme disease,” “neuroborreliosis,” “LNB,” “borreliosis.” We reviewed titles, abstracts, case reports, and full articles to assess their relationship with the research criteria. References reported in each article were also reviewed to identify additional study not found by initial search terms.

### Epidemiology of *Borrelia burgdorferi* infection

LB is increasing worldwide with ~300,000 new cases annually in the United States and 85,000 cases in Europe each year (28–30). Incidence of human LB in endemic areas of the United States ranges from 10 to 100 per 100,000 population with a peak of 134 per 100,000, reported in Connecticut in 2002 (4, 31, 32). The number of documented LB cases and the geographic distribution has expanded during the last two decades, from the Northeastern and North Central United States (4). LB is widespread also in Europe and the incidence for LB ranges from 20 to 80 per 100,000 in the Czech Republic, Germany, Latvia, the Netherlands, Poland, Switzerland, and Sweden, peaking to more than 100 per 100,000 in Austria, Estonia, Lithuania, and Slovenia (4, 29, 33, 34). Incidence of LB decreases southward, in Spain, France, Italy, and Greece with approximately 1 case per 100,000 (4, 29).

*B. burgdorferi* sensu stricto, *B. garinii*, and *B. afzelii*, are primarily responsible for human LB in different geographical regions presenting specific symptoms (34–38).

The genomes of *Borrelia* species consist of a set of circular and linear plasmids and a linear chromosome of ~900 kb ending with DNA sequences regulated by breakage and reunion reactions (39, 40). Different isolates show a variable number of plasmids depending on the species and affected by frequent reorganization (41–47). *B. burgdorferi* B31 strain harbors 10 circulars and 12 linear plasmids while *B. afzelii* B023 and *B. garinii* CIP 103362 have 6 linear and 2 circular plasmids and 4 linear and 1 circular plasmids in Fraser et al. (41), Casjens et al. (42), and Bontemps-Gallo et al. (47).

Most of the essential genes involved in metabolism or regulation are located in the linear chromosome while only a subset of genes encoding proteins required for growth and specific virulence factors are located on plasmids (41, 48–50).

*B. burgdorferi* sensu stricto is the predominant causative agent of LB, Lyme arthritis, and also LNB in the United States (51). Nevertheless, consistent differences in the ability to induce LB exist between *B. burgdorferi* sensu stricto subtypes suggesting that, neurotropism is an ability present only in a restricted subtype of *Borrelia* (52, 53). Different genotypes of *B. burgdorferi* sensu stricto diverge ecologically and epidemiologically, suggesting that genotype classification is relevant to understanding the basic biology of the spirochete (54–56). Studies conducted in endemic areas of the United States revealed that patients with disseminated infection were more likely infected by the RST1 strains of *B. burgdorferi* than with RST3 strains (57, 58). Moreover, dissemination of *B. burgdorferi* to blood or cerebrospinal fluid (CSF) was mostly related to *ospC* genotypes A, B, I, or K (58–62).

The distribution and relative frequency of infection by the different genospecies of *Borrelia* sensu latu vary across European regions. *B. burgdorferi* sensu lato comprises 20 different genospecies and this diversity correlates with the large variability in the clinical manifestations observed in LB (4, 13, 63). In the northern and eastern Europe *B. afzelii* is the most prevalent species, whereas in Western European countries *B. garinii* is the most common pathogen (4, 29). *B. afzelii, B. garini*, and the recently identified species *B. bavariensis* are major cause of LB and LNB in Europe (52, 64–68). The heterogeneity among *B. burgdorferi* sensu lato genospecies is linked to different geographical areas, which, in turn, correlates with the different clinical expression of human LB (69). For instance, *B. afzelii* induces prevalently skin infections, whereas *B. garinii* is in most cases neurotropic (5, 69). Other species, such as *B. lusitaniae* or *B. valaisiana*, have only occasionally been associated with human disease (70–72). In endemic areas of Europe was proposed that the variety of symptoms observed in children and adults with LNB correlated with the *B. burgdorferi sensu lato* genotype (73–75). Individuals with erythema migrans caused by *B. afzelii* and *B. garinii* showed distinct epidemiological and clinical characteristics. Indeed, erythema migrans caused by *B. garinii* were located prevalently on the trunk and less often on extremities, had shorter incubation and faster evolution, leading to frequent systemic symptoms, abnormal liver function test results than individuals with erythema migrans caused by *B. afzelii* (76, 77).

The genetic diversity observed in *B. burgdorferi*, at both inter- and intra-species level, is probably the reason for the multiple epidemiological and clinical presentation of these bacteria in humans (62, 78–80). A major role in maintaining the intraspecific genetic diversity of *B. burgdorferi* is the adaptation to multiple vertebrate hosts, which act as ecological niches for different genotypes (81, 82). Consequently, variations in the vertebrate host fitness may result in changes in the abundance of the more pathogenic species (83–85).

### Host invasion strategies of *Borrelia burgdorferi*

Colonization, dissemination and invasion of the tick vector and mammalian host by *B. burgdorferi* requires a complex temporal and spatial regulation of borrelial genes to adapt to environmental challenges. Transition of *B. burgdorferi* from the tick midgut to the hemolymph during a blood meal is an important step for bacterial diffusion through the salivary glands to a mammalian host (86). *Borrelia* possesses a sophisticated mechanism of gene regulation based on the two-component pathways HK1/Rrp1 and Rrp2-RpoN-RpoS, which regulate metabolism, antigenic variation, chemotaxis, and adhesion in a tissue- and temporal-specific manner in both the tick vector and mammalian host (48, 87). During the blood uptake *B. burgdorferi* expresses the outer surface proteins (Osp) A and B. These proteins mediate the adherence to the tick's gut by the binding to the tick receptor of OspA (TROSPA), thus facilitating the subsequent transmission into the mammalian host (88–90). Infection of the mammalian host requires the migration of the spirochetes from the midgut to the salivary glands of the tick. After the blood uptake into the midgut of the tick, the production of OspA and OspB decrease while *ospC* is expressed in conjunction with many other genes controlled by the RpoN, RpoS, and Rrp2 system (90–96).

OspC is required to establish the early phase of *B. burgdorferi* infection in mammalian host and to promote evasion from the innate immune defenses (96–98). Different studies revealed that the OspC mutant strains are unable to establish infection in mice, suggesting a protective role of this protein against host innate defenses (96, 99–104). Nevertheless, to escape from the host immune system, the expression of *ospC* decreases within 2–3 weeks after infection in response to anti-OspC antibodies in mice (105, 106). In addition to OspC, *B. burgdorferi* hides other important immunogenic surface proteins (107). In particular, OspA, which stimulates neutrophils and a strong inflammatory response mediated by interleukin (IL)-1β, tumor necrosis factor (TNF)-α, and IL-6, is highly expressed in the tick gut but it is rapidly downregulated in the host (108–112). OspA-positive strains of *B. burgdorferi* penetrate the host, but are unable to establish an infection (113). Similarly, *Borrelia* strains isolated from mice 4 days after infection, were found to be OspA negative suggesting that this protein is not expressed in the early phase of the host infection (114, 115).

The expression of different proteins, including OspC, ErpP, ErpA, ErpC, and enolase is required to readily immobilize host plasminogen on spirochetal surface, facilitating efficient dissemination (116–118). Plasminogen is a glycoprotein produced by the liver and abundant in the plasma and in certain tissues (119). Conversion of plasminogen to active plasmin is promoted by proteolytic activation induced by either tissue-type plasminogen activator (tPA) and/or urokinase-type plasminogen activator urokinase (uPA). Plasmin is responsible for intravascular fibrinolysis and contributes to numerous physiological and pathological processes, including tissue remodeling, cell migration, thrombolysis, wound healing, and cancer progression (120, 121). Invasive forms of *B. burgdorferi* are known to expresses multiple plasminogen-binding surface proteins that likely assist pathogen dissemination through host tissues (120, 122). Enolase is an integral enzyme of the glycolysis and gluconeogenesis pathways, and a multifunctional protein found in both prokaryote and eukaryotes (123). In eukaryotic cells, surface enolase acts as a plasminogen receptor in certain tumor cells (123, 124). Similarly, this enzyme is also localized on the cell surface of different microorganisms including *B. burgdorferi* (118, 125–127). The surface-localized enolase acts as a plasminogen receptor contributing to spirochetal survival in feeding ticks (118). Although dispensable for infection, plasminogen is required for dissemination in ticks, and its absence is associated with a decreased spirochetemia in plasminogen-deficient mice (128). Surface-associated plasmin on *B. burgdorferi* degrade fibronectin, which is an important component of the ECM, laminin and vitronectin (129, 130). *B. burgdorferi* also induces the release of host matrix metalloproteases 9 (MMP-9) and MMP-1, and plasmin-coated *B. burgdorferi* activates pro-MMP-9, leading to degradation of basement membranes (131).

*B. burgdorferi* exhibits a specific affinity for the CNS as demonstrated by the presence of spirochetes in the human CSF within 14–18 days after the tick bite (18, 132, 133). From the initial site of entry in the skin the spirochetes can reach the CNS either through the bloodstream or, alternatively, by the peripheral nerves (114).

Hematogenous dissemination from the tick bite on the skin to the CNS is a key pathogenetic event in LNB (114). However, it has been proposed that, at least for *B. garinii* which is mostly responsible for LNB in Europe, spirochetes can pass along the peripheral nerves (114). To penetrate the brain, spirochetes must first cross the blood-brain barrier reaching the brain microvascular endothelium and astrocytes (134). This barrier is composed by the brain microvascular endothelial cells (BMEC), astrocytes, basement membrane, pericytes, and neurons. The BMEC are firmly held together by tight junctions, presenting with a reduced transcytotic vesicles and an absence of fenestrae. All of these elements contribute to reduce the transport of solutes defending the brain from most pathogens or toxic agents (135, 136). Invasion of the blood-brain barrier by *B. burgdorferi* is still a matter of debate. Some studies suggest that *Borrelia* uses a paracellular route of translocation (134, 137), although other evidences suggest a possible transcellular passage of the spirochetes (138). Neurotropic *B. burgdorferi* strains showed the activation of the host plasminogen system, MMPs, and calcium signaling pathway to facilitate an efficient translocation through the blood-brain barrier (120, 134, 139). Compelling evidence suggest that *spirochetes* can adhere to murine neural and glial cell lines, primary neural cells, and primary rat brain cultures (140). In addition, *in vitro* studies show that *B. burgdorferi* can promote an intracellular invasion of human fibroblast, umbilical vein endothelial, synovial, neuronal, and glial cells without affecting the cell viability. This suggests that spirochetal cellular invasion may provide a mechanism for immune evasion and disease pathogenesis (140–142).

### Lyme neuroborreliosis

A common clinical and pathological manifestations of LNB in Europe is painful lymphocytic meningoradiculitis also known as Bannwarth syndrome, frequently accompanied by CSF signs of inflammation (13, 14, 143). The early manifestation of LNB generally appears within 2–18 weeks after infection (13, 143). The clinical description of painful meningoradiculitis was first reported in 1922, but the etiology remained unknown till the isolation of spirochetes by Burgdorfer in 1982 and the isolation in 1984 of spirochetes from the CSF of a patient with Bannwarth syndrome (144–146).

In addition to Bannwarth syndrome, other important neurological symptoms of the early stages of LNB include meningitis, meningeal perivascular, and vasculitic lymphoplasmocytic infiltrates, neuritis, and in rare acute LNB cases encephalitis and myelitis (143, 147). CNS vasculitis are rare in LNB, affecting mainly the large/medium-sized vessels and are associated with ischemia and stroke (19, 148). However, in European patients with LNB the mortality rate is comparable to that of the general population. Nevertheless, LNB is associated with increased risk of hematological and non-melanoma skin cancers (149).

Treatment with conventional intravenous antibiotic therapy, leads, in most cases, to a gradual improvement of the symptoms after several weeks or months, accompanied frequently by a normalization of CSF findings (150, 151). However, <2% of patients treated for LNB experience late neurological manifestations that persist months or years after *B. burgdorferi* infection (14, 143). The clinical symptoms of late LNB include several neurological and psychiatric symptoms such as meningoradiculitis, encephalomyelitis, chronic meningitis, and cerebral vasculitis (152–154). The presence of depressive states was described in 26–66% of patients with late LNB together with psychosis, schizophrenia, hallucinations, paranoia, anorexia nervosa, obsessive-compulsive disorder, and dementia (155–163). A frequent manifestation appearing in the late stage of LNB is the chronic vascular damage, clinically characterized by recurrent stroke or transient ischemic attacks (153, 154). Other distinctive findings in patients with late LNB are inflammatory CSF changes (CSF pleocytosis and elevated total protein content) and the presence of specific *B. burgdorferi* intrathecal antibody (150, 151).

The clinical outcome of antibiotic treatment of either early or late manifestations of LNB may include progression to a chronic form characterized by nonspecific and persistent fatigue, arthralgia, myalgia, musculoskeletal, and cognitive symptoms. This condition, frequently defined as posttreatment Lyme disease syndrome (PTLDS), can be intermittent or persistent, lasting for at least six or more months after completion of antibiotic treatment (143, 164).

Specific diagnostic criteria for PTLDS proposed by the Infectious Disease Society of America relies on the objective proof of previous LB, the presence of subjective symptoms that compromise function in daily life, and the absence of clinical evidences for another underlying illness (7). However, those criteria have rarely used in clinical studies, contributing to confusion and controversy about the clinical significance of PTLDS syndrome (7). The frequency of PTLDS among patients with LB varies largely, ranging from 0 to 50%, depending upon differences in study design and enrollment criteria (165, 166). A long-term follow-up study of patients with early presentation of erythema migrans and treated with antibiotics at the time of diagnosis showed an excellent rate of remission, with only 4% of patients remaining symptomatic during follow-up evaluation (167). Conversely, other trials reported rates of PTLDS ranging from approximatively 10–20% (168). Nevertheless, in the community medical practice, where prompt LB diagnosis and treatment are not common, PTLDS rates may reach 50% (169, 170). Notably, xenodiagnoses demonstrated the presence of *B. burgdorferi* DNA in a patient with PTLDS, despite repeated cycles of antibiotic treatments (171). *B. burgdorferi* DNA was detected in mice after prolonged (up to 12 months) treatment with antibiotics despite the persistence of non-cultivable *bacteria*. Moreover, the study revealed *B. burgdorferi* DNA and the presence of RNA transcripts of multiple spirochetal genes in host tissues (172). These findings suggest that *B. burgdorferi* persist within the host indicating that the immune system and antimicrobial treatment may not be effective at eradicating *B. burgdorferi*. This may contribute to antibiotic-refractory arthritis, as observed in a murine model in which spirochetal antigens, but not infectious spirochetes, were recovered near cartilage for extended periods after LB therapy (173).

According to the guidelines of the European Federation of Neurological Sciences and the Infectious Diseases Society of America, treatment with beta-lactams antibiotics, like ceftriaxone, penicillin, or cefotaxime, or oral doxycycline for 14–21 days is recommended for the treatment of LNB (7, 25, 174). Intravenous administration of ceftriaxone is often recommended for the treatment of Lyme meningitis. Oral treatment with doxycycline demonstrated to be as effective as ceftriaxone for Lyme meningitis in adults in Europe, although not recommended as first-line therapy in the United States (175). Nevertheless, four NIH-sponsored trials aimed at assessing the administration of antibiotic treatment in patients with persistent unexplained symptoms despite previous antimicrobial treatment of LB indicated that the new treatment cycle provides little if any clinical benefit (176). A randomized, double-blinded, placebo-controlled trial conducted in Europe, in patients with persistent symptoms attributed to Lyme disease showed that longer-term antibiotic treatment did not have a better outcome as compared with shorter-term treatment (177).

### Biofilm production and antimicrobial tolerance in *Borrelia burgdorferi*

*B. burgdorferi* can switch from motile cellular forms into several defensive morphological forms such as round bodies, stationary phase, persister cells, and biofilm (23, 24, 178–182). Transition between different morphologies represents an adaptation strategy to survive in unfavorable environmental conditions, including pH variations, nutrient starvation, host immune system attacks, or the presence of antimicrobial agents (21, 23, 24, 172, 179, 181).

**Figure 1:**
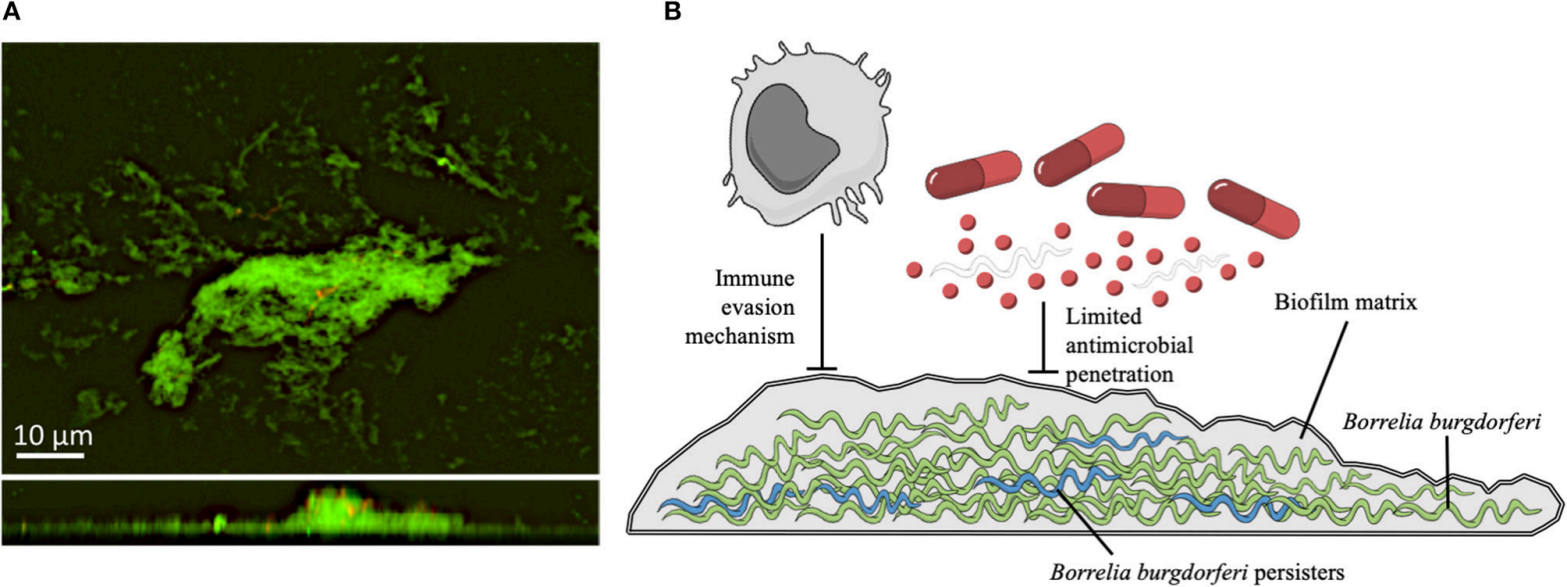
**(A)** Confocal microscopy images of *B. burgdorferi* B31 strain (American Type Tissue Collection 35210) biofilms. Upper panel show the X-Y planes (top view), while the lower panel show the Z section (side view). The sample was stained with *BacLight* Live/Dead (Invitrogen Life Technologies, Carlsbad, CA, USA) (188). Representative images of biofilms developed on polystyrene pegs following 72 h incubation at 37°C. Spirochetes were grown in a μ-Slide 8-well system (ibidi, Germany) and cultured in BSK-H medium containing 6% rabbit serum (Sigma-Aldrich). **(B)** Schematic representation of a *B. burgdorferi* biofilm. The biofilm matrix produced by spirochetes (green) provides shelter from host defenses, and reduces the diffusion of antibiotics. Persister forms of *B. burgdorferi* (in blue), exhibit multidrug tolerance and are likely responsible for the recalcitrance of chronic LB. The illustration is adapted from Mind the Graph (https://mindthegraph.com) under the Creative Commons License.

Notably, within the biofilm, bacteria are physically joined together producing a matrix, characterized by the presence of an extracellular polymeric substance (EPS) composed by polysaccharides, proteins, and extracellular DNA (183). Bacterial biofilms are intrinsically more resistant to environmental agents and antimicrobials than the corresponding planktonic counterpart and this can lead to chronic and recurrent infections (184–186). *In vitro* and *in vivo* studies revealed that both *B. burgdorferi* sensu stricto and sensu lato (*B. afzelii* and *B. garinii*) aggregates, but not free-floating spirochetes, present typical markers found in the EPS of other pathogenic bacteria such as sulfated mucins, non-sulfated mucins (mainly alginate), extracellular DNA and calcium (22, 23, 187). *B. burgdorferi* biofilm is also characterized by the presence of a distinctive architecture with channel-like elements that in mature biofilm are required for oxygen and nutrient diffusion and waste removal (22, 23, 187). Biofilm formation by *B. burgdorferi* follows the same evolution described for other bacteria. Initially, individual spirochetes adhere to biotic or abiotic surfaces forming microcolonies, coated by the EPS. From this point, *Borrelia* aggregates expand undergoing changes in the growth rate, gene expression and structural rearrangements in the EPS components (22, 23). The rapid rearrangements occurring within the biofilm matrix, culminate in a complex three-dimensional structure with common traits observed among *Borrelia* genera (Figure 1) (23, 189). The existence of biofilm-like structures was further found in human skin biopsies obtained from patients with borrelial lymphocytoma, a common manifestation of LB in Europe, revealing the presence of *Borrelia*-positive aggregates characterized by mucopolysaccharides, especially alginate (22). Other reports demonstrated that *Borrelia* DNA is deposited in the Alzheimer brain showing structural similarities between spirochetal aggregates and the profiles of amyloid plaques in patients with Alzheimer disease (188, 190). Nevertheless, the specific contribution of biofilm to borrelial persistent infections remain unclear. Besides, additional *in vivo* studies showed the presence of *Borrelia* aggregates in the midguts of naturally-infected nymphs during their blood meal (191). These results strongly suggest that biofilm may contribute to the spirochetal successful transmission to the mammalian host and to the ensuing disease manifestations (191).

Biofilm production in *Borrelia* requires the modulation of a complex array of signaling processes which allows spirochetes to communicate with the surrounding environment. The RpoN–RpoS alternative sigma factor and the LuxS quorum-sensing pathways, which are involved in several cellular functions in response to environmental stresses (pH and temperature variations, high osmolarity, oxidative stress, high cell density, nutrient starvation, host infection), participate in biofilm production in *B. burgdorferi* (22, 192). The RpoN–RpoS pathway, also known as the σ^54^-σ^S^ cascade, regulates adaptive changes in *B. burgdorferi* during the transition between the tick vector and mammalian host (91, 95). The RpoN–RpoS pathway relies on the activity of RpoN (σ^54^), which controls the transcription of RpoS (σ^S^) through the binding to a canonical −24/−12 RpoN-type promoter sequence (95, 193). The activation of the σ^54^-σ^S^ cascade, in turn, is modulated by a bacterial enhancer-binding protein (bEBP)/σ54-dependent activator (Rrp2) in concert with BosR (91, 95, 193–198). After the activation, RpoS acts as a global gene regulator controlling the expression of over 100 different genes involved in stress responses, host infection and survival, including biofilm formation (87, 95, 182).

Mutant strains of *B. burgdorferi* lacking RpoN, RpoS, presented a less compact biofilm with loose and dispersed small aggregates compared to wild-type strains (182). Notably, all mutants expressed *Borrelia* biofilm markers such as alginate, extracellular DNA, and calcium, although they showed significantly higher sensitivity to low MIC dose of doxycyline (0.1 μg/ml) than the wild-type strain (182). In addition, the quorum sensing (QS) molecules LuxS also contributes to *B. burgdorferi* biofilm. The QS signaling system is a cell-to-cell communication mechanism, shared by different bacteria, which is based on the release of small molecules called autoinducers (AI) in environment (199). LuxS pathways regulate biofilm formation in various ways according to bacterial species and environmental conditions (183). Specifically, *luxS* mutant strains of *Streptococcus gordonii* and *Porphyromonas gingivalis*, which are two important components of dental plaque, are unable to produce a mixed-species biofilm (200). Besides, in *Helicobacter pylori* the presence of *luxS* mutation leads to a more efficient biofilm formation than the wild type whereas a *luxS* mutant of *Streptococcus mutans* shows an altered biofilm structure (183, 201–203). *B. burgdorferi* significantly increases transcription of *luxS* during transition from ticks to mammalian hosts where it is involved in the regulation of several genes such as *vlsE, erpA*, and *ipLA7* (204–207). *luxS* mutant strains in stationary cultures of *B. burgdorferi* showed a higher tendency to form smaller and looser aggregates and a greater sensitivity to antibiotics than the wild-type counterpart (182).

Although antibiotic treatment resolves most of clinical manifestations of LB, persistent forms occur in ~10% of patients after treatment for erythema migrans disease (208, 209). The long-term persistence of symptoms and failure of the antibiotic therapy are reminiscent of chronic biofilm-associated infections. Biofilm aggregates display an enhanced tolerance to various antibiotics, which, conversely, are effective against the planktonic spirochetes and round body forms of *B. burgdorferi* (210). In particular, doxycycline and amoxicillin were found to effectively kill the motile spirochete forms *in vitro*, but failed to completely remove *B. burgdorferi* in biofilms (20, 21, 24, 26, 210–214). High throughput screens of *B. burgdorferi* identified several promising Food and Drug Administration (FDA)-approved drugs that have excellent anti-persister activity (24, 181, 213, 215). Among them, daptomycin, which is a lipopeptide targeting bacterial cell membranes, clofazimine, carbomycin, sulfa drugs such as sulfamethoxazole, and certain cephalosporins such as cefoperazone, showed higher activity against *B. burgdorferi* persister cells resulting more effective than doxycycline or amoxicillin (213). Although the combination of these drugs was found to be active against *B. burgdorferi* persisters, they showed poor activity when used individually (24). Daptomycin was found to be the most active antibiotic when combined with doxycycline plus either beta-lactams like cefoperazone or carbenicillin or alternatively with clofazimine (212). Daptomycin in combination with doxycycline and cefoperazone was found to be able to completely eradicate *B. burgdorferi* persisters, revealing a durable killing activity that was not achieved by any other drug combinations (212). These results where further supported by prospective randomized clinical studies which failed to demonstrate significant beneficial effect of additional prolonged therapy with doxycycline, amoxicillin or ceftriaxone in monotherapy, in patients with Lyme encephalopathy and post-treatment symptoms of Lyme disease (176, 215).

In addition to biofilm formation, the ability of *Borrelia* to localize intracellularly in the host has been proposed as a mechanism which might favor chronic or persistent infection and may contribute in reducing the efficacy of antibiotics. However, *Borrelia* predominantly occupies the extracellular matrix, and the antibiotics recommended for the treatment of LB are first-line drugs in several intracellular infections (216, 217). Doxycycline and azithromycin are commonly used for the treatment of *Mycoplasma, Chlamydia*, and *Legionella*, while ceftriaxone is effective against *Salmonella* and *Neisseria*, and amoxicillin is used to treat *Listeria* infections (217, 218). Nevertheless, biofilm production by extracellular bacteria and intracellular localization of *Borrelia* are not mutually exclusive and may both participate in supporting chronic bacterial persistence in the host.

On the other hand, a polymicrobial infection is a frequent occurrence in ticks (219, 220). Chronic and persistent forms of Lyme have been also associated to infections caused by *Babesia* spp. and *Anaplasma phagocytophilum, Bartonella henselae*, or other minor pathogens (217, 219, 221). This condition may add a further level of complexity to the clinical and therapeutic management of LB since it may lead to inappropriate diagnoses and apparent failure of the antibiotic treatment targeted exclusively against *Borrelia*. However, the real clinical relevance of these coinfections is unclear and requires further, more in depth evaluation.

## Concluding remarks

LNB is the most dangerous manifestation of Lyme disease. Although the early antimicrobial treatment is effective in the majority of patients, persistent forms are relatively common. The mechanisms underlying chronic LNB and other persistent forms of Lyme are unknown. Patients who have late manifestations of LB generally show a slower response to therapy with incomplete resolution. Persistent *Borrelia* infection requires prolonged antimicrobial treatment, with limited and controversial clinical efficacy. Recent evidences suggest that the antibiotic resistance and the reoccurrence of LB are associated with biofilm-like aggregates, which allow *Borrelia* spp. to resist to adverse environmental conditions. Several promising FDA-approved drugs have been shown to have excellent anti-persister activity when used in combination while their use in monotherapy regimens showed a poor effectiveness. This notion should be taken into careful consideration for the clinical management of Lyme Disease in order to prevent long-term complications.

In preliminary studies by the clinical Biofilm Ring Test® (cBRT), we found that *Borrelia* is able to readily produce biofilm within 24–48 h. Diagnostic procedures such as the cBRT, which allow for a rapid biofilm measurement may represent very useful tools for clinical applications (222, 223), since the rapid identification of biofilm-producing *Borrelia* strains, may help identify forms of LB which are at risk of chronicity (224). Further, characterization of *Borrelia* biofilm as well as the ensuing inflammatory process will likely provide novel insight to better understand the mechanism(s) concurring to LNB pathogenesis and may offer new therapeutic targets for intervention.

## Author contributions

Conceived and designed the study: ED, IC, LT, FP, and FE. Performed the confocal microscopy analysis: ED, IC, MP, GP, and MG. All authors analyzed data. Wrote the paper: ED, IC, LT, VB, GD, ET, and FE. All the authors read and approved the final version of the manuscript.

### Conflict of interest statement

The authors declare that the research was conducted in the absence of any commercial or financial relationships that could be construed as a potential conflict of interest.
